# Catch me if you can: viral nucleic acids to host sensors

**DOI:** 10.3389/fimmu.2025.1632283

**Published:** 2025-07-28

**Authors:** Yohan Jung, Harmony Grainger, Shizhuo Yang, Sohaumn Mondal, Kiven Erique Lukong, Kristen Conn, Yuliang Wu

**Affiliations:** ^1^ Department of Biochemistry, Microbiology and Immunology, University of Saskatchewan, Saskatoon, SK, Canada; ^2^ Department of Veterinary Microbiology, Western College of Veterinary Medicine, University of Saskatchewan, Saskatoon, SK, Canada

**Keywords:** sensor, DNA virus, RNA virus, innate immunity, adaptive immunity

## Abstract

The 2002 movie *Catch Me If You Can* is a cat-and-mouse story in which Frank Abagnale Jr. successfully conned his way into several high-profile jobs while evading capture by FBI agent Carl Hanratty. Similarly, after entering host cells, viruses interact with or hijack host cellular machinery to replicate their genetical materials and assemble themselves for the next round of infection. Analogous to an FBI agent, host cells have numerous molecular “detectives” that recognize viral nucleic acids (NAs). These include RIG-I, MDA5, LGP2, TLR3, TLR7, TLR8, DHX36, DICER1, PKR, OAS1, ZAP, and NLRP1/6 for viral RNA, as well as cGAS, TLR9, AIM2, IFI16, IFIX, Ku70, MRE11, RNA polymerase III, hnRNPA2B1, LRRFIP1, DAI, DHX9 and DDX41 for viral DNA. However, much like the brilliant Frank Abagnale Jr., viruses have developed various strategies to evade host cellular surveillance—for example, by sequestering or modifying viral NAs and inhibiting or degrading host sensors. In this review, we will summarize the host sensors identified so far, discuss the latest understandings of the various strategies employed by viruses, and highlight the challenges associated with drug development to target virus or host factors. Considering recent global health challenges such as the COVID-19 pandemic and undergoing measles outbreak, understanding virus-host interactions at the molecular and cellular levels remains essential for the development of novel therapeutic strategies.

## 1 Introduction

German philosopher Friedrich Nietzsche said, “That which does not kill us makes us stronger”. The COVID-19 pandemic not only accelerated the development of mRNA-based therapeutics but also provided a unique opportunity on a global scale and real-time to deepen our understanding of viral infection mechanisms. Viral entry begins with the attachment of the spike (S) protein of SARS-CoV-2 to the angiotensin-converting enzyme 2 (ACE2) receptor on human cells (1). The S protein is cleaved by host cell proteases, such as transmembrane protease serine 2 (TMPRSS2) (2), facilitating the fusion between the virus and the host cell. After fusion, SARS-CoV-2 RNA genome is released into the cytoplasm, where it hijacks the host cell’s transcriptional and translational machinery to replicate its genetic material and synthesize viral proteins. These components are assembled into new virions, transported to the cell surface in vesicles, and released through exocytosis to infect other cells. Simultaneously, host cells deploy a variety of immune strategies to counter viral replication; however, viruses continually evolve mechanisms to evade these defenses. This dynamic interaction between viral evasion and human immune responses constitutes an evolutionary “arms race”, characterized by ongoing adaptation on both sides.

**Figure 1:**
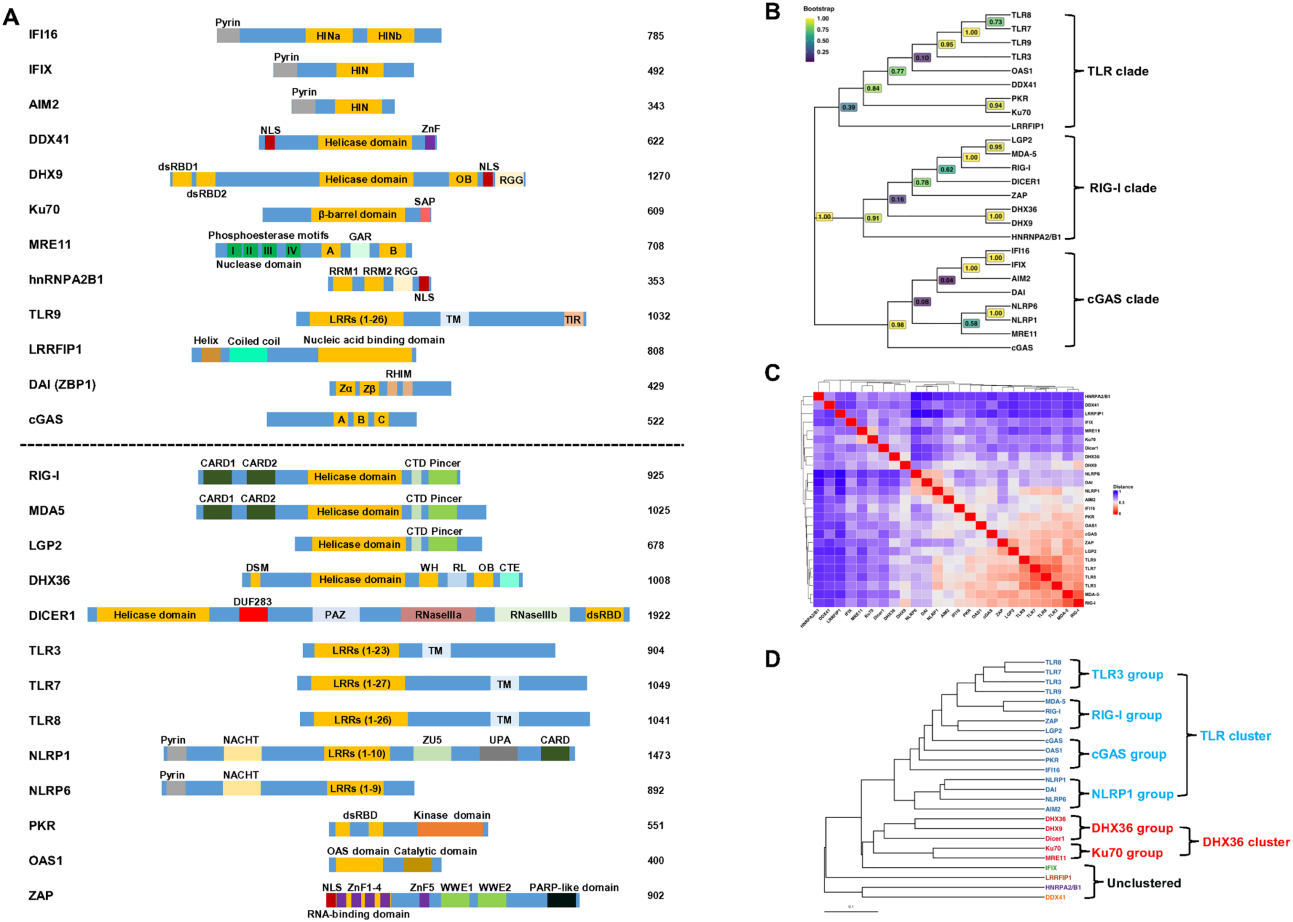
Alignment and classification of human viral nucleic acid sensors. **(A)** The alignment of human DNA sensors (above dash line) and RNA sensors (below dash line). The DNA or RNA binding domains are highlighted in orange, and an individual protein’s functional domain(s) and motif(s) are indicated. IFI16 contains a pyrin domain and two HIN domains (HINa and HINb) that bind DNA; IFIX has a pyrin domain and a HIN domain that binds DNA; AIM2 has a pyrin domain and a DNA binding domain HIN-200; DDX41 contains a helicase core domain; DHX9 contains dsRNA binding domain 1 and 2, a helicase core domain, an OB-fold (oligosaccharide-binding) and a RGG (repeated arginine-glycine-glycine) domain; Ku70 has a central DNA-binding domain and a SAP (SAF-A/B, Acinus, and PIAS) domain; MRE11 has two DNA binding domains (A and B) and a GAR (Glycine-Arginine-Rich) motif; hnRNPA2B1 contains two quasi-RRM (RNA recognition motif) domains and a RGG; TLR3, 7, 8, 9 have various LRRs (leucine-rich repeats), a transmembrane domain (TM), and TIR (Toll/Interleukin-1 receptor) domain; LRRFIP1 contains a helix, coiled-coil and a LRR domain; DAI (ZBP1) contains two Z-DNA binding domains (Zα and Zβ); cGAS has three DNA binding domains (A, B and C); RIG-I, MDA5 and LGP2 contain two CARD (Caspase Activation and Recruitment Domain, except LGP2), a helicase core domain, a C-terminal domain (CTD), and a Picer domain; DHX36 has a DSM (DHX36-specific motif), a helicase core domain, a WH (winged-helix) domain, RL (ratchet-like) domain, a OB fold, and a C-terminal extension; DICER1 has a helicase core domain, a domain of unknown function (DUF283), PAZ (Piwi-Argonaute-Zwille) domain, two RNase III domains (IIIa and IIIb), and a double-stranded RNA binding domain (dsRBD); NLRP1 and 6 have a Pyrin domain, NACHT (NAIP, CIITA, HET-E and TP-1) domain, and LRR, NLRP1 has additional ZU5 (ZO-1 and UNC5), UPA (UNC5, PIDD and Ankyrin domain) and a CARD; PKR has two dsRNA-binding domains and a kinase domain; OAS1 contains two dsRNA binding domains and a catalytic domain; ZAP has five zinc finger domain and two WWE domains. NLS, nuclear localization signal; NES, nuclear export signal. **(B)** Phylogenetic tree of human viral nucleic acid sensors. Protein sequences were retrieved from UniProt and aligned using the AlignSeqs function from the DECIPHER package in R (15). Positions with >40% gaps were trimmed prior to tree construction. The best-fitting substitution model (LG+G[4]+I) was identified using the modelTest function in the phangorn package for more accurate estimation of evolutionary distances, branch lengths, and topology. An initial neighbor-joining tree was constructed from a maximum likelihood (ML)-based distance matrix and optimized using the optim.pml function under the selected model. Bootstrap support values were calculated from 1,000 replicates using the bootstrap.pml function with topology optimization enabled. The final tree was midpoint-rooted and visualized using the ggtree package (16), with bootstrap values displayed on internal nodes using a viridis color scale. Branch lengths were suppressed to highlight evolutionary relationships and clade structure based on amino acid sequence homology. **(C)** Correlation heatmap of human viral nucleic acid sensors. Semantic data for Gene Ontology (GO) Biological Process (BP) terms were obtained from the org.Hs.eg.db annotation database using the godata function in the GOSemSim package (17). Pairwise semantic similarity scores between genes were computed using the mgeneSim function, yielding a score from 0 (functionally unrelated) to 1 (identical) for each gene pair. These similarity scores were then converted into a distance matrix (1 – similarity) and visualized as a clustered heatmap using the ComplexHeatmap package (18). Hierarchical clustering was performed using average linkage, and the same clustering was applied to both rows and columns. For interpretability, protein names are displayed along the axes instead of gene symbols. **(D)** Cluster dendrogram of human viral nucleic acid sensors. The tree was constructed from the same distance matrix used in the heatmap (C) and visualized using the ggtree package. Six clusters were defined based on cluster stability analysis performed with the ConsensusClusterPlus package. Branches and tip labels are color-coded by cluster assignment, with protein names displayed at the tips. The scale bar represents functional distance.

Host cells have evolved various defense mechanisms to detect and eliminate viruses, including physical barriers, immune responses, and cellular processes like autophagy, which degrades viral components. Among these defenses, pattern recognition receptors (PRRs) recognize pathogen-associated molecular patterns (PAMPs) present on viruses (3). These PAMPs, which include viral nucleic acids (NAs, RNA or DNA) and proteins, are recognized by an array of PRRs. For instance, endosomal Toll-like receptors (TLRs) and cytoplasmic RNA helicases, such as retinoic acid-inducible gene I (RIG-I) -like receptors (RLRs), initiate antiviral immunity by inducing the production of type I and III interferons (IFNs) along with inflammatory cytokines. Specific PRRs detect distinct viral components; for example, TLR4 recognizes viral envelope proteins, while C-type lectin receptors identify carbohydrate structures on the surface of pathogens, including the glycoproteins from human immunodeficiency virus (HIV), Dengue virus (DENV), and Ebola virus (EBOV). Specialized PRRs for NA sensing include RIG-I, MDA5, LGP2, TLR3, TLR7, TLR8, DHX36, DICER1, PKR, OAS1, ZAP, and NLRP1/6 for viral RNA, whereas cGAS, TLR9, AIM2, IFI16, IFIX, Ku70, MRE11, RNA polymerase III, hnRNPA2B1, LRRFIP1, DAI, DHX9 and DDX41 are key sensors for viral DNA (**Figure 1A**, **Table 1**). Collectively, these NA sensors orchestrate a robust antiviral response that enables host cells to counteract viral infection.

**Table 1:** Summary of viral nucleic acid sensors identified in humans.

Sensor	DNA viruses	RNA viruses	Location	Notes	References
TLR3^a^	dsDNA: HSV-1	dsRNA: rotavirus. (+) ssRNA: PV, SARS-CoV-2	Cytoplasm (endosome)		(31–34)
TLR7 and TLR8		(+) ssRNA: SARS-CoV-2, CV, EMCV. (–) ssRNA: IAV, MV, SeV, VSV	Cytoplasm (endosome)		(55, 56)
TLR9	dsDNA: MCMV, HSV-1	(+) ssRNA: Dengue virus, SARS-CoV-2	Cytoplasm (endosome)		(43, 58, 63, 64)
RIG-I (DDX58)		(+) ssRNA: JEV, DENV, EMCV, ZIKV, SARS-CoV-2. (-) ssRNA: NDV, SeV, VSV, IAV	Cytoplasm		(67, 261–263)
MDA5		(+) ssRNA: SARS-CoV-2, HCV, picornavirus family (EV-A71, CVB3, CVA21, EMCV)	Cytoplasm		(67, 199, 264, 265)
ZAP	dsDNA: MVA, HBV	(+) ssRNA: SFV, SINV, HTLV-1, JEV, ZIKV, SINV, CVB3, SARS-CoV-2, EV-A71, retrovirus HIV-1. (–) ssRNA: IAV, EBOV, MARV, SeV, NDV	Cytoplasm		(87, 266, 267)
LGP2 (DHX58)		(+) ssRNA: EMCV. dsRNA: BPEV	Cytoplasm		(76, 79)
cGAS	dsDNA: HSV-1, KSHV	(+) ssRNA: SARS-CoV-2, retrovirus HIV-1	Nucleus, cytoplasm, membrane		(99, 106, 107, 109)
OAS1		(+) ssRNA: SARS-CoV-2, WNV	Cytoplasm		(116)
PKR		(+) ssRNA: PV, EMCV, EV70, CVB3, CVB5, VSV. (–) ssRNA: MV, IAV, NDV, SeV	Cytoplasm		(119, 121, 122, 125)
IFI16	dsDNA: HSV-1, HCMV	(+) ssRNA: retrovirus HIV-1. (-) ssRNA: IAV/PR8	Nucleus, Cytoplasm	Mechanisms for detection of ssRNA viruses remain unknown	(129, 133, 136, 137)
NLRP1		(+) ssRNA: SFV	Cytoplasm		(138)
DAI (ZBP1)	dsDNA: VACV, NDV, HSV-1, MCMV	(-) ssRNA: IAV	Nucleus, Cytoplasm		(140, 141, 143, 268)
NLRP6		dsRNA: rotavirus. (+) ssRNA: MHV	Cytoplasm		(139)
AIM2	dsDNA: VACV, MCMV, HCMV	(+) ssRNA: SARS-CoV-2. (-) ssRNA: IAV	Cytoplasm		(144, 147, 154, 155)
DHX36	dsDNA: HSV-1, HBV	(-) ssRNA: NDV, IAV	Nucleus, Cytoplasm		(120, 165, 172)
DHX9	dsDNA: HSV-1, MHV-68	dsRNA: Reovirus. (-) ssRNA: IAV	Nucleus, Cytoplasm		(165–167)
DICER1		(+) ssRNA: ZIKV, HIV-1, EV-A71 (-) ssRNA: IAV	Cytoplasm		(176–178, 180)
Ku70	dsDNA: HSV-1, -2, HBV	(+) ssRNA: retrovirus HTLV-1	Cytoplasm	Primarily form Ku70/Ku80 heterodimeric protein in NHEJ, but can act independently as a sensor for viral DNA/RNA	(185, 188)
MRE11	dsDNA: HSV-1		Cytoplasm	Primarily as MRE11-RAD50-NBS1 (MRN) complex in DNA repair, but MRE11 can function alone as a dsDNA sensor	(189)
IFIX	dsDNA: HSV-1, VACV		Cytoplasm	Signaling cascade remains unknown	(195, 196)
LRRFIP1		dsRNA: VSV. (+) ssRNA: SARS-CoV-2	Cytoplasm		(197, 199)
hnRNPA2B1	dsDNA: HSV-1		Nucleus		(201)
DDX41	dsDNA: HSV-1	(+) ssRNA: retrovirus MLV	Nucleus, Cytoplasm		(202–204)
RNA polymerase III	dsDNA: HSV-1, HCMV, EBV, VZV	(+) ssRNA: SINV		Containing 17 subunits, and DNA-binding is mediated by the core subunits RPC1, RPC2 and RPABC1.	(21–24)

a, Please see the details of abbreviations in glossary.

Based on their genetic materials, viruses are broadly classified into DNA or RNA viruses. DNA viruses can be single-stranded (ssDNA) or double-stranded (dsDNA); examples of dsDNA viruses include herpesviruses, adenoviruses, and poxviruses, while ssDNA viruses include parvoviruses and anelloviruses. Similarly, RNA viruses are categorized as either single-stranded (ssRNA) or double-stranded (dsRNA). ssRNA viruses are further divided into positive-sense (+) ssRNA viruses, such as coronaviruses, retroviruses, picornaviruses, and flaviviruses, and negative-sense (–) ssRNA viruses, including orthomyxoviruses and rhabdoviruses. Examples of dsRNA viruses include sedoreoviruses and picobirnaviruses. To establish successful infection, viruses have evolved diverse evasion strategies to escape detection by host cell sensors. These strategies include sequestration and modification of viral NAs, inhibition of immune signaling pathways, degradation of host sensors, and exploitation of immune checkpoints. Collectively, these mechanisms allow viruses to circumvent the host immune response and propagate effectively.

Although several comprehensive reviews have addressed host nucleic acid sensors and their biological functions (4–6), viral evasion strategies (7, 8) and individual viruses (9, 10), an updated and integrative understanding of the dynamic interplay between viral NAs and human sensors remains elusive. In this review, we summarize the host sensors identified in humans to date, with emphasis on the sophisticated strategies employed by viruses to evade these immune sensors. By focusing on viruses known to infect humans and the corresponding host sensors, we will provide insight into how these interactions facilitate viral replication and propagation within the host.

## 2 Virus infection

Viruses are highly adept at infiltrating host cells, commandeering cellular machinery, and replicating within the host. This stepwise process enables them to spread and survive by exploiting the host’s resources. The initial stage of infection involves the attachment of a virus to the surface of a host cell, mediated by interactions between viral surface protein(s) and host cell receptor(s). Once attached, viruses have various ways to enter the host cell.

After entry, the virus releases its genetic material from the outer layer (capsid) in a process called uncoating, which exposes its genome within host cells to initiate viral protein production and genome replication. Positive-sense (+) ssRNA viruses, such as poliovirus (PV) and SARS-CoV-2, have genomes that function directly as mRNA for protein synthesis (11, 12). In contrast, negative-sense (–) ssRNA viruses, such as influenza virus, carry genomes that are complementary to mRNA and must first be transcribed into (+) ssRNA (mRNA) by a viral RNA-dependent RNA polymerase (RdRp) to serve as a template for translation. dsRNA viruses, such as rotavirus, transcribe mRNA from their genomes using their RdRp within protective capsids. Retroviruses, such as HIV, use their reverse transcriptase to convert RNA into complementary DNA (cDNA) within the capsid. The cDNA is then released into the nucleus, where it is integrated into the host genome by the viral integrase and subsequently the integrated DNA provirus is transcribed into RNA. DNA viruses commonly transport their genomes from the capsid to the nucleus to utilize the host cell’s replication machinery during their replication cycle. For example, the dsDNA genome of adenoviruses is released into the nucleus, where it serves as a template for replication and transcription. Similarly, ssDNA viruses, such as parvoviruses, deliver their genomes to the nucleus, where the ssDNA is first converted into a dsDNA intermediate by host DNA polymerases, which then serves as the template for viral transcription and replication (13).

New viral particles are assembled by packaging replicated genetic material with newly synthesized viral proteins. These virions are then released from the host cell through lysis, membrane budding, or exocytosis. Efficient assembly and release of virions are critical for viral propagation and transmission to new host cells.

## 3 Human defense against viral infection

Initial barriers against viral entry are physical, such as the skin, mucosal membranes, and stomach lining, which prevent foreign pathogens from entering the body. For instance, to lower the risk of rotavirus infection, intestinal epithelial cells act as a physical barrier and produce mucus, cytokines, and chemokines (14). Upon viral infection, host cells activate a variety of defense mechanisms, including both innate and adaptive immune responses. The innate immune response acts as the first line of defense, relying on conserved elements of pathogens to rapidly destroy invaders. The initial innate immune response is triggered when viral components such as RNA, DNA, or intermediate products are detected by the host, inducing the expression of IFNs and other pro-inflammatory cytokines. PRRs, either constitutively expressed (e.g., TLR9 and RIG-I) or upregulated (e.g., TLR3 and MDA5), can identify PAMPs, which are conserved structures found on pathogens. PRR activation provides immunoprotective advantages by initiating signalling pathways that connect innate and adaptive immune responses. Cytokines, chemokines, and IFNs are the main products of these pathways, with IFNs being especially well-known for their antiviral qualities. Type I IFNs are crucial in inducing an antiviral state, as they are secreted by infected host cells in response to virus infection and can induce the expression of hundreds of interferon-stimulated genes (ISGs), which have antiviral functions and block viral replication. Cytokines also enhance the antigen-presenting function of antigen-presenting cells (APCs), as well as the antiviral function of adaptive immune cells. Natural killer (NK) cells are essential in the innate immune response to viral infections. When triggered, they produce cytotoxic granules that include granzymes and perforin, which create holes in the target cell membrane and cause apoptosis. Furthermore, NK cells can elicit apoptosis through interaction with death receptors such as TRAIL or Fas.

In contrast, the adaptive immune response is activated by the innate immune system, targeting specific antigens on pathogens and relies on a coordinated interaction between APCs, T, and B lymphocytes. This interaction facilitates specific immune responses against pathogens, forming long-term immunologic memory, and maintaining immune system balance. T cells play three key roles: activate other immune cells, detect and destroy infected cells, and retain a record of the antigen, enabling faster response upon reinfection. T helper cells activate B cells, in a process called T cell-dependent humoral immune response to secrete antibodies. Another mode of activation for B cells is in a T-cell-independent manner, without the presence of antigen presentation. Memory B cells allow for rapid response and neutralizing antibodies in subsequent infections.

The efficacy and specificity of the immune response require a multitude of immune modulators such as sensors, inflammatory cytokines, B cells, T cells, with responses that vary significantly based on viral entry, replication, and spread. A critical aspect of the immune response, especially in viral infection, is sensor-mediated detection. PRRs are key sensors that recognize exogenous NAs from invading viruses. These receptors are found on both innate and adaptive immune cells, and include TLRs, RLRs, C-type lectin receptors, and NOD-like receptors (NLRs), enabling them to detect and respond to viral infections effectively.

## 4 Viral nucleic acid sensors in human cells

A total of 26 host sensors have been identified to detect viral DNA and RNA (**Table 1**, **Figure 1A**). To understand their evolutionary relationships, we performed a phylogenetic analysis. Using their amino acid sequences (except RNA polymerase III that contains 17 subunits), DECIPHER (15) and ggtree (16) R packages, phylogenetic analysis categorized these 25 sensors into three major clades: TLR, RIG-I and cGAS (**Figure 1B**). While the phylogenetic tree reflects evolutionary relatedness based on sequence similarity, it does not fully capture the functional similarities of these sensors. Therefore, we assessed their biological functional similarities based on Gene Ontology (GO) Biological Process (BP) annotations (17). Pairwise semantic distance matrix was derived from GO-based semantic similarity scores (ranging from 0 for functionally unrelated to 1 for identical), and hierarchical clustering was performed on this matrix. The distance matrix (**Figure 1C**) was then visualized using the ComplexHeatmap package (18) and a corresponding dendrogram (**Figure 1D**) based on the same functional distances was generated using the ggtree package (16). This functional analysis identified six distinct clusters: a large TLR-associated cluster comprising 16 proteins, a DHX36-associated cluster with five members, and four unclustered proteins with limited functional similarity to other groups. We will discuss these clusters accordingly. Although discussed separately, it is important to note that host nucleic acid sensors often exhibit crosstalk during viral infections. For example, RIG-I and cGAS have been shown to act synergistically in the context of HIV infection (19), while RIG-I and IFI16 display antagonistic interactions during herpes simplex virus type 1 (HSV-1) infection (20).

RNA polymerase III is a key cytosolic sensor of viral nucleic acids, primarily recognizing DNA viruses such as HSV-1, human cytomegalovirus (HCMV), Epstein-Barr virus (EBV), and varicella zoster virus (VZV) (21–24), as well as the RNA virus Sindbis virus (SINV) (22). However, it is not classified into any specific clade or cluster, as it comprises 17 subunits and current classification tools are unable to analyze multi-subunit protein complexes. In addition, we did not discuss uncommon sensors such as DDX23 (25), SNRP200 (26), NLRC3 (27) and RPSA (28), or those whose function is debatable, such as DDX60 (29, 30).

### 4.1 TLR-associated cluster

TLR-associated cluster can be further divided into four groups: TLR3, RIG-I, cGAS and NLRP1 (**Figure 1D**).

#### 4.1.1 TLR3 group

**Figure 2:**
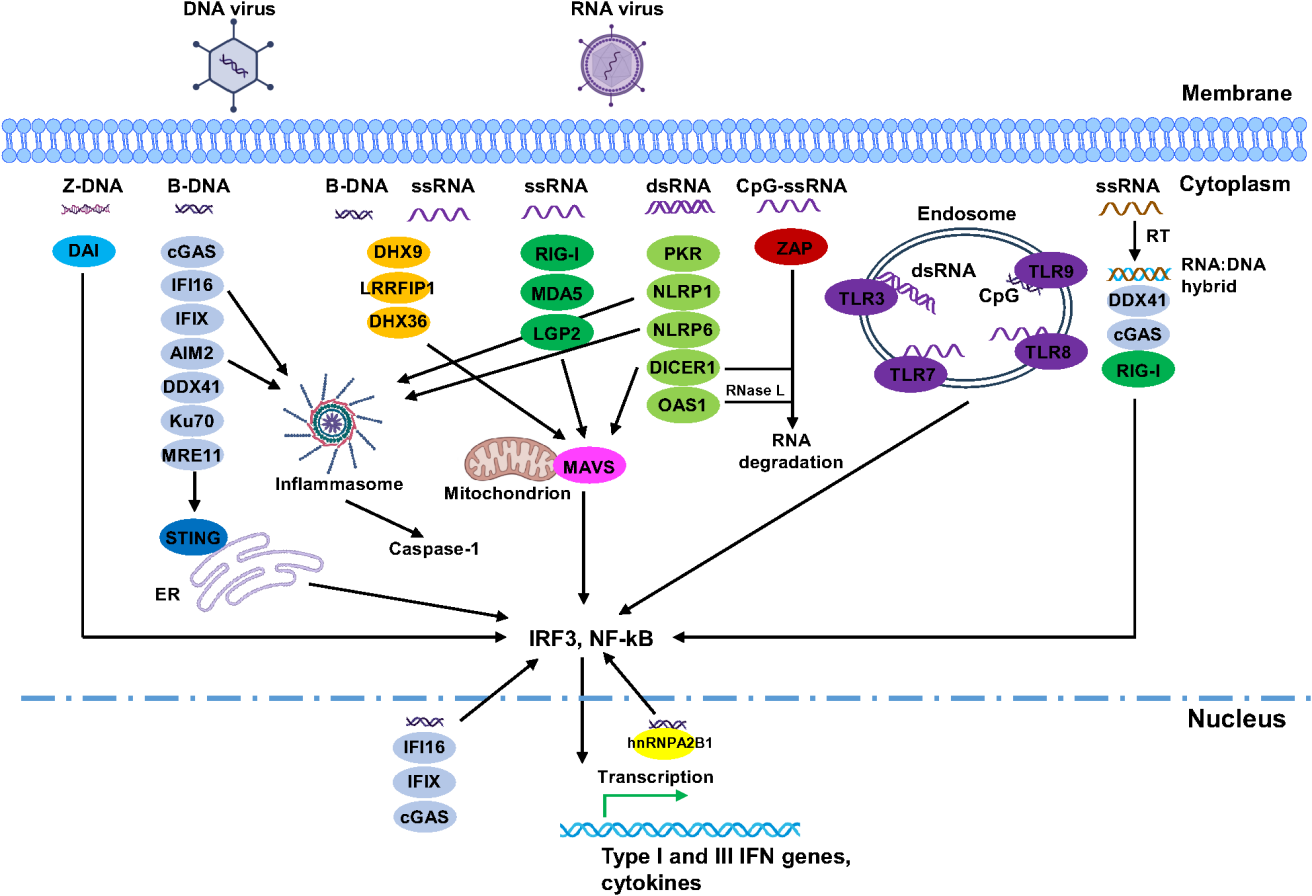
Summary of the interplay between viruses and human viral nucleic acid sensors. For simplicity, only DNA and RNA viruses were shown as examples. Focusing was mainly on different forms of viral nucleic acids and their sensors, downstream pathways were simplified. In addition, many of these sensors can recognize various forms of nucleic acids and are involved in different signalling pathways. Images were created by BioRender.

The TLR3 group is composed of TLR3, TLR7, TLR8 and TLR9 (**Figure 1D**). To date, 10 human TLRs (TLR1-10) have been identified. These receptors are localized into the cell membrane and endosomes and are expressed in a wide range of cell types, including immune cells and non-immune cells. Among the TLRs found in endosomes, TLR3 recognizes dsRNA, TLR7 and TLR8 recognize ssRNA, and TLR9 recognizes CpG DNA (**Figure 2**). Structurally, TLRs consist of an N-terminal ectodomain with leucine-rich repeat (LRR) motifs responsible for recognizing PAMPs/DAMPs (damage-associated molecular patterns), a transmembrane domain, and a C-terminal cytoplasmic Toll/IL-1R (TIR) domain (**Figure 1A**).

TLR3 primarily senses dsRNA viruses (31–34). Studies have found that 40–50 bp dsRNA can bind TLR3 and induce its dimerization, leading to basal activation (35), whereas a robust immune response requires longer dsRNA of at least 90 bp (36–39). Upon binding to dsRNA, TLR3 dimerizes and signals through the adapter protein TRIF (TIR-domain-containing adapter-inducing IFN-β, also called TICAM1). This TLR3-TRIF-mediated signaling predominantly activates IRF3/7 to induce the production of type I IFN, particularly IFN-β; while also, to a lesser extent, activates NF-κB and AP-1 to promote the production of cytokines (e.g., IL-6, IL-12, IL-1α, and CSF-3) (40). TLR3 also recognizes (+) ssRNA viruses, such as West Nile virus (WNV) (41), DENV (42), PV (32), SARS-CoV-2 (34), and dsDNA viruses, including murine cytomegalovirus (MCMV) (43) and HSV-1 (31, 44). The precise mechanisms by which TLR3 recognizes ssRNA or dsDNA viruses remain unclear, but it is likely that dsRNA structures, including secondary stem-loop formations in ssRNA viral genomes and dsRNA intermediates generated during viral replication (45, 46) activate TLR3. Similarly, many DNA viruses produce dsRNA intermediates during transcription, possibly arising from the bidirectional transcription within their genomes (46). Notably, TLR3 has been shown to recognize the stem-loop structures of PV (32) and the non-coding RNA transcripts EBERs from EBV, which are transcribed by RNA polymerase III (24). TLR3 activation is influenced by the topology, secondary, and tertiary structures of dsRNA regions (32). Therefore, it is plausible that TLR3 recognizes dsRNA structures derived from ssRNA or dsDNA viruses, initiating immune responses.

The TLR7 subfamily consists of TLR7, TLR8, and TLR9, which share highly homologous structures and activate similar antiviral mechanisms upon detecting single-stranded nucleic acids. These receptors contain a Z-loop located between LRR14 and LRR15 in the extracellular domains, which must be cleaved for activation. All three sensors utilize the adaptor MyD88 to relay signaling, leading to the activation of NF-κB, AP-1, and IRF3/5/7 (47). TLR7, TLR8, and TLR9 possess two binding sites that contribute to subtle differences in ligand recognition. TLR7 has one site for guanosine and its derivatives (e.g., 2',3'-cGMP) and uridine-containing ssRNA at another (48, 49). TLR8 recognizes uridine at one site and short ssRNA at another (50). Recent evidence demonstrated that endonuclease RNase T2 is important for TLR7 and TLR8 ligands (51, 52). Specifically, RNase T2 generates guanosine 2′,3′-cyclic monophosphate-terminated RNA fragments, and PLD exonuclease further releases the terminal 2′,3′-cyclic guanosine monophosphate (2′,3′-cGMP), which becomes the ligand for TLR7 (52). RNase T2 preferentially cleaves ssRNA between purine and uridine residues, which generates catabolic uridine as a TLR8 ligand (51). TLR9 binds two types of DNA: one site recognizes CpG DNA, while another recognizes 5′-xCx DNA (cytosine at the second position from the 5′ end) (53, 54). TLR7 and TLR8 recognize a wide variety of ssRNA viruses, including Coxsackie virus (CV), Encephalomyocarditis virus (EMCV), influenza A virus (IAV), measles virus (MV), Sendai virus (SeV), vesicular stomatitis virus (VSV), and SARS-CoV-2 (55, 56), activating antiviral immune responses.

The discovery that TLR9 recognizes unmethylated CpG bacterial DNA and initiates immune responses was first reported by Dr. Shizuo Akira’s group (57). For viral infections, TLR9 is not only limited to recognizing CpG ssDNA but also detects dsDNA viruses with unmethylated CpG motifs in their genomes, such as MCMV, adenovirus, and HSV-1/2 (43, 58–60). Prior to viral infection, TLR9 is expressed in the endoplasmic reticulum, upon infection, it is translocated into endosomes, where it binds CpG-containing viral DNA and signals through MyD88 to activate antiviral inflammatory pathways (59, 61). Moreover, BAD-LAMP regulates the trafficking of TLR9 to LAMP1^+^ late endosomes in human plasmacytoid dendritic cells, promoting NF-κB activation and the production of pro-inflammatory cytokines. In contrast, TLR9 can also signal from a distinct VAMP3^+^/LAMP2^+^/LAMP1^-^ endolysosomal compartment, which primarily enhances type I interferon production (62). In addition, two independent studies demonstrated that during viral infections, such as DENV or SARS-CoV-2, TLR9 recognizes mitochondrial (mt) DNA that is released into the cytosol, leading to NF-κB activation and an antiviral immune response (63, 64). Notably, the study on TLR9-mediated responses in DENV infection confirmed the colocalization of TLR9 and DNA in DENV-infected dendritic cells (DCs) (63).

#### 4.1.2 RIG-I group

The RIG-I group comprises RIG-I, MDA5, ZAP and LGP2 (**Figure 1D**). The RLR subfamily of PRRs includes retinoic acid-inducible gene I (RIG-I, also known as DDX58), melanoma differentiation-associated protein 5 (MDA5), and laboratory of genetics and physiology 2 (LGP2, also known as DHX58). These cytoplasmic RNA sensors share a central DExD/H-box helicase core domain, which contains a DECH motif, and a carboxy-terminal domain (CTD) responsible for foreign RNA substrate recognition. Both RIG-I and MDA5 feature two amino-terminal caspase activation and recruitment domains (CARDs) that mediate signal transduction to downstream effectors. In contrast, LGP2 lacks these CARDs but still binds RNA with high affinity, with a preference for blunt-ended dsRNAs over internal dsRNA regions or RNA overhangs (65) and acts as a regulator of RLR signaling (66). Despite structural homology (**Figure 1**), the RLRs exhibit slight differences in their RNA recognition preferences.

The CTD of RIG-I detects viral RNA bearing 5′ triphosphate or 5′ diphosphate specifically (66), playing a crucial role in the antiviral response against RNA viruses, such as paramyxoviruses, influenza virus, and Japanese encephalitis virus (67). Upon infection, viral RNA interacts with RIG-I through the CTD, triggering a conformational change that exposes the N-terminal CARD, allowing RIG-I oligomerization and interaction with the adaptor protein mitochondrial anti-viral-signaling protein (MAVS). CARD-dependent interaction with MAVS activates transcription factors IRF3 and NF-κB, promoting the expression of type I IFNs and ISGs. The spatiotemporal regulation of RIG-I involves coordinated control of its activation, localization, and signaling dynamics. In the absence of infection, RIG-I resides in the cytoplasm in an auto-repressed conformation. Upon activation by viral RNA, RIG-I translocates to mitochondrial-associated membranes, where it interacts with the adaptor protein MAVS. This signaling is amplified through K63-linked ubiquitination by E3 ligases such as TRIM25 or Riplet, which stabilizes RIG-I’s association with MAVS (68). To prevent excessive inflammation, RIG-I activity is negatively regulated by several mechanisms, including deubiquitinating enzymes such as CYLD and USP21 (69, 70), autophagic degradation (71), and suppression by SOCS proteins (72). RIG-I recognizes short dsRNA (10–300 bp) with 5′-triphosphate or 5′-diphosphate ends. In contrast, MDA5 preferentially binds dsRNA longer than 1 kb without end specificity. Like RIG-I, MDA5 binds viral dsRNA through its CTD, oligomerizes to form filaments along the viral dsRNA, and then activates MAVS through its CARDs (73).

Initially, LGP2 was thought to act as a negative regulator, interfering with RIG-I and MDA5 RNA recognition (74). However, recent studies indicate that LGP2 serves as a positive regulator, enhancing viral RNA recognition and MDA5 activation (75, 76), while negatively regulating RIG-I (77, 78).The exact mechanisms behind this remain under investigation, but LGP2’s ATPase activity facilitate binding to diverse dsRNA species and works synergistically with MDA5 to improve antiviral efficiency (65, 76, 79). Further studies show that LGP2 is recruited to MDA5-dsRNA filaments, promoting filament formation along the dsRNA and stabilizing MDA5’s interaction with dsRNA during (+) ssRNA EMCV and dsRNA Bell Pepper Endornavirus (BPEV) infections in HEK293T cells (76). Additionally, LGP2 induces a conformational change in MDA5, exposing its CARDs completely, which activates MAVS and leads to the activation of immune response genes, including type I IFNs. Like RIG-I, MDA5 is a cytoplasmic sensor that interacts with MAVS on the outer mitochondrial membrane upon recognition of viral dsRNA. However, unlike RIG-I, MDA5 typically responds during the later stages of infection and is negatively regulated by factors such as LGP2 (80).

Zinc-finger antiviral protein (ZAP), also known as zinc-finger CCCH-type antiviral protein 1 (ZC3HAV1) or poly (ADP-ribose) polymerase family member 13 (PARP13), functions as a sensor for a variety of RNA and DNA viruses. Its N-terminus contains an RNA-binding domain (RBD) with four zinc finger domains (ZnF1-4), while the middle of the protein contains the fifth zinc finger (ZnF5) and two tandem WWE modules (WWE1 and WWE2) at the C-terminus (**Figure 1A**). The RBD is responsible for binding ssRNA, and the WWE2 pocket specifically binds poly (ADP-ribose) (PAR). ZAP was originally identified as an antiviral factor restricting MLV via a cDNA expression library screening in 2002 (81). Recent CLIP-seq and ZAP’s structure with RNA showed that ZAP interacts with CpG dinucleotides in RNA (82, 83). Consistent with its structure, ZAP is a sensor for a variety of RNA viruses, such as hepatitis E virus (HEV) (84), Ebola virus, chikungunya virus, and hepatitis B virus (HBV) (85, 86). However, DNA viruses, such as HBV (85), HCMV (87), and vaccinia virus (VACV) (88), have been reported as targets of ZAP as well. Similar to other sensors, ZAP exist in four isoforms: S, M, L, and XL, which have distinct antiviral roles (85, 89).

#### 4.1.3 cGAS group

The cGAS group is made up of cGAS, OAS1, PKR and IFI16 (**Figure 1D**). Compared with other groups, four members of this group have longer functional distances, indicating their divergent functions. Human cyclic GMP-AMP synthase (cGAS) was discovered by Dr. Zhijian Chen’s lab a decade ago (90). It consists of 522 amino acids, with a long unstructured, positively charged N-terminal and a catalytic C-terminal (**Figure 1A**). The C-terminus contains two key domains: the nucleotidyltransferase (NTase) core domain, which is essential or cGAS enzymatic function, catalyzing GTP and ATP into cGAMP, and the Mab21 domain, which features a Zn ribbon motif [H(X_5_)CC(X_6_)C] that mediates DNA binding and ensures specificity for B-form dsDNA (91–93). The C-terminus has three DNA binding sites (A, B, and C) composed of positively charged amino acid residues that interact with negatively charged DNA backbone (92–95). Collectively, both the positively charged C- and N-termini of cGAS form multivalent interactions with the negatively charged DNA in a sequence-independent manner, promoting the formation of liquid-like droplets containing activated cGAS (96). As a DNA sensor, cGAS detects viral dsDNA from HSV-1 (97, 98) and KSHV (99) and produces cGAMP. Then, cGAMP activates the STING-TBK1-IRF3- and -NF-κB-mediated inflammatory pathways to induce the expression of type I IFNs and proinflammatory cytokines (97–100).

Initial, studies reported that cGAS is localized in the cytoplasm, where it detects viral dsDNA (98, 99). More recent findings have identified additional localizations, including the plasma membrane (101) and nucleus (97, 102–104). At the plasma membrane, cGAS is anchored to the inner leaflet via phosphatidylinositol 4,5-bisphosphate, keeping it in an inactive state (101). In the nucleus, cGAS is tethered to chromatin, also maintained in an inactive state (97, 102–105). Notably, this sequestration prevents the indiscriminate activation of cGAS by endogenous DNA while positioning it to optimally detect viral DNA (97, 101). Upon viral infection, cGAS is released, allowing it to recognize viral DNA and trigger initiate immune signaling (97, 101, 104).

cGAS also plays a critical role in RNA virus infections. In SARS-CoV-2 infection, cGAS binds to genomic dsDNA released from the nucleus or mtDNA due to infection-induced cellular stress, activating the STING-mediated pathway to trigger an antiviral response (106). In HIV-1 infection, cGAS detects primary reverse transcribed cDNA, antisense ssDNA with stem-loop structures, during early infection. This activates STING-mediated signaling, triggering an innate immune response (107–110).

Oligoadenylate synthetases (OASs) play key roles in the innate immune defense against viral dsRNA in the cytosol of vertebrates. In humans, there are four members in this family: OAS1, OAS2, OAS3, and OASL. Each of them contains a polymerase beta (pol-β)-like nucleotidyl transferase domain (also known as OAS domain), varies in the number of this domain. OAS1 has one pol-β domain (OAS domain I, DI), OAS2 has two pol-β domains (DI and DII), and OAS3 has three pol-β domains (DI, DII, and DIII). While OAS1–3 have one enzymatical active OAS domain, additional OAS domains in OAS2–3 remain inactive (DI in OAS2, DI and DII in OAS3). In contrast, OASL contains a single pol-β domain at its N-terminus and two consecutive ubiquitin-like domains in its C-terminus but lacks enzymatic activity entirely. Interestingly, OAS proteins do not contain a canonical dsRNA-binding domain or motif; however, dsRNA binding is achieved through electrostatic interactions and hydrogen bonds between the dsRNA and positively charged residues in a groove located on the surface of OAS proteins (111–114). OAS1 has a bilobular structure composed of N-terminal and C-terminal lobes, with a positively charged groove between them responsible for binding two minor grooves of dsRNA ≥18 bp, leading to a conformational change and activation of the protein (111, 112, 115). In contrast, the presence of additional inactive domains in the N-terminal region of OAS2 and OAS3 requires longer dsRNA for activation, with OAS2 (two OAS domains) binding to four minor grooves of dsRNA and requiring > 35 bp (114), while OAS3 (three OAS domains) requires dsRNA > 50 bp for activation (113, 115). Higher activation of OAS proteins is observed with longer dsRNA, OAS3 showing greater activation than OAS1 in response to equal amounts of dsRNA > 60 bp, highlighting the importance of dsRNA length in distinguishing the self from non-self target (114, 115). During viral infection, OAS1 recognizes viral dsRNA and catalyzes the production of 2′-5′-oligoadenylate, a secondary messenger that activates RNase L, an enzyme responsible for ssRNA cleavage (**Figure 2**). Studies have shown that OAS1 binds dsRNA structures (stem-loops) in the 5′-UTR of SARS-CoV-2, effectively blocking viral replication (116, 117). Whereas an isoform of OAS1, p46, confers strong antiviral activity against picornaviruses, flaviviruses, and SARS-CoV-2 (118).

Protein kinase R (PKR) is a cytosolic dsRNA sensor that contains an N-terminal dsRNA binding domain with two dsRNA-binding motifs (dsRBM1 and dsRBM2) and a C-terminal kinase domain (**Figure 1A**). PKR detects viral dsRNA, including those from IAV, newcastle disease virus (NDV), enteroviruses (PV, EV70, CVB3, CVB5), SeV and MV (119–122), as well as endogenous dsRNA (i.e., non-coding (nc) RNAs, mtRNA transcripts) released into the cytoplasm (122, 123). Upon binding to dsRNA, PKR undergoes dimerization and phosphorylation, which in turn phosphorylates eIF2α, blocking the translation of viral mRNA and inhibiting viral replication (124). Previous studies have demonstrated PKR’s critical role in activating RLRs, such as RIG-I and MDA5, within stress granules (SGs), thereby facilitating antiviral responses during infections with IAV, NDV, PV, and SeV (119–121, 125). Based on these findings, SGs were considered essential for PKR and RLR activation, as their impairment or absence significantly reduced the activation of these viral sensors and subsequent antiviral responses. However, recent studies suggest that PKR and RLR activation and localization are independent of SGs (122, 126, 127). One study revealed that rather than serving as sites for viral RNA sensing and PKR/RLR activation, SGs regulate antiviral responses by preventing excessive activation of RIG-I, PKR and OAS signaling, as well as apoptosis, while also intrinsically restrict viral replication and spread in a RLR/PKR-independent manner (127). Moreover, other studies have indicated that PKR does not localize to SGs or processing bodies (P-bodies) but forms distinct autonomous clusters in response to dsRNA stress and interacts with dsRNA-binding proteins (122, 126). Notably, dsRNA stress induces the formation of dsRNA-induced foci (dRIF), which contain dsRNA, PKR, and various dsRNA-binding proteins, including ADAR1, Stau1, DHX9, NLRP1, and protein activator of PKR, with the timing and localization of dRIF formation strongly correlating with PKR activation (126). To prevent aberrant PKR activation, a recent CRISPR screening revealed that PACT cooperates with ADAR1 to suppress PKR activation from self-dsRNAs in uninfected cells (128).

IFI16 (Interferon Gamma Inducible Protein 16) contains an N-terminal PYD domain and two DNA-binding HIN domains, known as HINa and HINb (**Figure 1A**). It is located in the nucleus and serves as a DNA sensor to detect viral DNA (129–133). Upon binding viral DNA through its HIN domains, IFI16 activates the STING-mediated inflammatory pathway, leading to the induction of type I IFNs and proinflammatory cytokines (133–135). In addition to inducing IFN signaling, IFI16 plays a role in epigenetic regulation. Studies highlight that IFI16 binds to HSV-1 gene promoter regions, promoting the formation of repressive heterochromatin (marked by H3K9me3), thereby suppressing HSV-1 gene transcription and replication (129–131, 135). Moreover, IFI16 binds to these promoters and inhibits the recruitment of RNA Pol II and transcription factors, such as TBP and Oct1, further suppressing HSV-1 gene transcription (130). Besides its role in sensing DNA viruses, IFI16 is implicated in recognizing RNA virus, such as IAV (136) and HIV-1 (137). A recent study demonstrated that IFI16 directly binds IAV (A Puerto Rico/8/1934 H1N1, PR8) via its HINa domain, leading to enhanced RIG-I transcription and interaction with RIG-I through its PYD domain, creating a synergistic antiviral response (136).

#### 4.1.4 NLRP1 group

The NLRP1 group consists of NLRP1, DAI, NLRP6, and AIM2 (**Figure 1D**). NLRP1 and NLRP6 share similar structural organization, including a central nucleotide-binding and oligomerization (NACHT) domain flanked by an N-terminal pyrin (PYD) domain and C-terminal LRRs. Both proteins function as viral sensors, though they recognize different viral components. NLRP1 detects pathogen-derived enzymes, such as proteases, and senses dsRNA. In contrast, NLRP6 primarily detects viral dsRNA.

NLRP1 senses the viral RNA of Semliki Forest virus. Mechanistically, it binds dsRNA through its LRR domain, leading to a conformational rearrangement that facilitates immune activation (138). NLRP6 interacts with dsRNA directly and undergoes liquid liquid phase separation both *in vitro* and in cells. This phase separation is crucial for activation of the NLRP6 inflammasome, a process further supported by investigation in mouse models infected with mouse hepatitis virus or rotavirus (139). Notably, the human genome encodes 14 distinct NLRP family members (NLRP1–14), many of which play key roles in inflammasome activation.

DNA-dependent activator of interferon-regulatory factors (DAI), also known as ZBP1 or DLM-1, is a critical nucleic acid sensor in the innate immune system. Originally identified as a cytosolic DNA sensor, DAI detects foreign DNA and activates type I IFN production, along with other innate immune responses (140). Mechanistically, DAI binds dsDNA through its Z-DNA binding domains, facilitating the recruitment of IRF3 and TBK1 to enhance immune signaling. Interestingly, recent studies have highlighted DAI’s role as an RNA virus sensor. It was found to recognize IAV genomic RNA (Z-RNA), binding to RIPK3 and recruiting MLKL and RIPK1, ultimately activating cell death pathways (141). In addition, DAI can interact with RNA transcripts derived from DNA viruses like murine cytomegalovirus (MCMV) and VACV. This interaction triggers necroptosis through the RIPK3 pathway (142, 143).

AIM2 (Absent in melanoma 2) consists of a single PYD domain and a single HIN domain (**Figure 1A**). As a cytosolic innate immune receptor, it recognizes both microbial and self-dsDNA in the cytoplasm. AIM2 can recognize a variety of dsDNA viruses, such as VACV (144, 145), MCMV (144), EBV (146), and human cytomegalovirus (HCMV) (147). During viral infection, the positively charged HIN domain of AIM2 interacts with the negatively charged dsDNA (≥ 80 bp in length) in a sequence-independent manner, through hydrogen bonds at both the major and minor grooves, leading to the formation of AIM2-ASC-caspase-1 inflammasomes (148). This assembled inflammasome cleaves pro-caspase-1 into active caspase-1, which matures proinflammatory cytokines IL-1β and IL-18 and cleaves gasdermin D (GSDMD), forming pores in the cell membrane that induce pyroptosis (149, 150). As a result, pyroptotic cell death releases cytosolic contents, including IL-1β and IL18 into the extracellular space, amplifying the inflammatory response (149, 151, 152). Notably, AIM2 is also implicated in antiviral responses against RNA viruses, such as enterovirus (EV-A71) (153), IAV (PR8) (154) and SARS-CoV-2 (155).The precise mechanism of how AIM2 recognizes RNA viruses remain unclear, However, researchers propose that mtDNA release or the uptake of self-DNA from dead cells may contribute to AIM2 activation (154, 155).

### 4.2 DHX36-assocaited cluster

DHX36-associated cluster can be further divided into two groups: DHX36 and Ku70 (**Figure 1D**).

#### 4.2.1 DHX36 group

The DHX36 group have DHX9, DHX36 and Dicer1 (**Figure 1D**), which all contain a helicase domain (**Figure 1A**). DHX9 (also known as RHA) and DHX36 (RHAU or G4R1) are DExD/H-box helicases characterized by the conserved DExD/H motif, specifically the DEIH motif for both. They are localized in both the nucleus and the cytoplasm (156, 157), where they regulate mRNA translation by unwinding G-quadruplex (G4) RNA structures in the 5' UTR of mRNA (158, 159). In addition to G4-RNA, both helicases unwind G4-DNA and other non-canonical nucleic acid structures, such as R-loops and D-loops (160–162). Structurally, DHX9 and DHX36 share a common core helicase domain of two RecA-like domains (RecA1 and RecA2) and an oligonucleotide/oligosaccharide-binding (OB)-like fold in the C-terminus (**Figure 1A**). However, there are slight differences in their N- and C-terminal regions and binding preferences. DHX9 has two dsRNA-binding domains (dsRBD1 and dsRBD2) in its N-terminus, while its C-terminus features a HA2 domain and glycine rich (G-patch) region, which enhances RNA duplex unwinding (163, 164). In contrast, DHX36 possesses a glycine-rich element followed by the DHX36-specific motif (DSM) in its N-terminus, which is critical for high affinity binding to G4 structures in DNA and RNA, as well as RNA duplexes (161, 162). Functionally, DHX9 preferentially binds and unwinds G4-RNA, R-loops, G4-DNA, D-loops, and RNA forks in descending efficiency, favoring RNA-containing duplexes with 3’ overhang tails (164); whereas DHX36 exhibits a broader preference for G4-RNA, G4-DNA, and RNA duplexes (161, 162).

Besides their roles in nucleic acid metabolism, DHX9 and DHX36 function as cytosolic viral sensors. They recognize a range of viral nucleic acids, including single-stranded CpG ODNs and dsDNA viruses such as HSV-1 and MHV (Murine Hepatitis Virus)-68 (165, 166). They also detect dsRNA viruses (e.g., reovirus) and ssRNA viruses (e.g., NDV, IAV) (120, 167), leading to the induction of type I IFNs and proinflammatory cytokines. The mechanisms underlying DHX9 and DHX36 viral recognition are well elucidated. The helicase core of DHX9 (RecA domains, HA domain, and OB-like fold) binds ssRNA (163, 164) and interacts with CpG ODNs via its OB-like fold (165). Its N-terminal RBDs recognize dsRNA structures in RNA viruses, further amplifying immune signaling (163, 164, 167). Similarly, DHX36 binds ssDNA/ssRNA through its helicase core and C-terminal domains (161), with CpG ODNs interacting with its DEAH motif (165). The conserved 5’-β-hairpin region within the helicase core and the OB-like fold in the C-terminus facilitate recognition of RNA duplexes and G4 structures (162). Additionally, the DSM motif in the N-terminus and the OB-like fold of DHX36 enhances binding to G4 structures, RNA duplexes (161, 162), and dsRNA forms of ssRNA viruses (120). Multiple studies have reported the presence of G4 structures in the viral genomes and mRNA transcripts (168–172). This raises the possibility that viral G4 structures may activate DHX9 and DHX36 during infection. Though further studies are needed to elucidate the mechanisms of G4-mediated immune responses by these helicases.

Human Dicer1 is a large protein (~219 kDa), mainly known as an endoribonuclease, not a helicase though containing a helicase domain. It is found in both the nucleus and cytoplasm. During viral infection, Dicer1 processes viral dsRNA or viral pre-microRNAs (pre-miRNAs) into small interfering RNAs (siRNAs) or miRNA (173, 174), which then bind to target viral mRNA or viral RNA genomes, promoting RNA interference (RNAi) (175). Dicer1 is crucial for processing dsRNA substrates into small RNAs to promote RISC-mediated RNAi and this process has been well established in numerous studies. Studies have shown that Dicer1 is responsible for the biogenesis of miRNAs and siRNAs derived from viral dsRNA, including those from HIV-1 (176), IAV/delNS1 (177), and EV-A71 with defective 3A protein (178), as well as dsRNA hybrid duplexes of HIV-1 genome and human tRNA (179). These small RNAs, incorporated into the RISC complex, bind viral mRNA or the sense strand of viral RNA genomes, to suppress viral gene expression and inhibit replication. Recently, the Reis e Sousa group identified a naturally occurring, alternatively spliced isoform of human Dicer1, named antiviral Dicer (aviD), which lacks the Hel2i domain due to the absence of exons 7 and 8 (180). Previous studies from other groups revealed that while the N-terminal helicase domain of Dicer negatively regulates its ability to process dsRNAs, its absence in mutant forms significantly enhances the processing of dsRNA substrates (181, 182). Consistent with these findings, the study demonstrated that aviD significantly enhances the processing of dsRNA substrates from SARS-CoV-2 and Zika viruses (ZIKV), generating higher levels of compared to canonical Dicer1 while retaining comparable miRNA production (180). This ultimately leads to improved RNAi in human and mouse stem cells. Not all small RNAs suppress viral replication through RNAi, but they can impede antiviral responses in humans. For instance, during SARS-CoV-2 infection, Dicer1 processes the stem‐loop structure of ORF7a to produce two miRNAs, CoV2-miR-07a.1 and CoV-2-miR-07a.2, in infected human cells (183). However, these miRNAs suppress host innate immune responses by binding to the 3′ UTR of ISGs and facilitating RISC-mediated degradation of these genes in infected cells.

#### 4.2.2 Ku70 group

The Ku70 group has Ku70 and MRE11 (**Figure 1D**), two well-known proteins involved in DNA double-stranded breaks (DSB) repair. Human Ku70 is a 609 amino acid nuclear protein consisting of three domains: an N-terminal von Willebrand factor domain, a β-barrel DNA-binding domain in the core, and a C-terminal SAP domain (**Figure 1A**). It exists as a heterodimer with Ku80, a complex that is essential for the stability of both proteins. Together, the Ku70/Ku80 heterodimer associates with DNA-dependent protein kinase catalytic subunit (DNA-PKcs) to form the holoenzyme DNA-PK. As a key component of DNA-PK, the Ku70/80 dimer plays a critical role in repairing DSB through non-homologous end joining (NHEJ). In addition to its role in DNA repair, Ku70 also functions as a cytosolic viral DNA sensor, activating innate immune responses against DNA viruses. Upon viral DNA infection, such as HSV-1 (184), HSV-2 (185), and HBV (186), Ku70 translocates from the nucleus to the cytoplasm, where it activates the STING-mediated signaling pathway. This leads to the activation of IRF1, IRF3, and IRF7, promoting the production of type I (IFN-α/β) and type III (IFN-λ1) IFNs (184–187). Further investigation revealed that acetylation in Ku70’s NLS domain promotes cytoplasmic translocation, enhancing the production of IFN-λ1 and inhibiting viral replication (184). Ku70 is also involved in the recognition of RNA viruses. One study revealed that Ku70 recognizes (+) ssRNA retrovirus human T-cell lymphotropic virus 1 (HTLV-1), leading to STING activation and the phosphorylation of NF-κB and IRF3, subsequently inducing an antiviral response (188).

MRE11 is best known for its role in DNA repair as part of the MRE11-RAD50-NBS1 (MRN) complex, which detects and initiates DSB repair. However, it also functions as a cytosolic exogenous dsDNA sensor by recognizing sequences such as HSV-1 and IFN stimulatory DNA, and stimulates type I interferon by regulating STING trafficking (189). More recently, it was reported that MRE11 is essential for cGAS-STING activation (190). Mechanically, cGAS is constitutively inhibited by high-affinity binding to the nuclear chromatin histone H2A-H2B acidic patch (AP) region on the nucleosome disk face that prevents its oligomerization and activation in response to dsDNA (91, 102, 105, 191–194), now MRN complex releases cGAS from nucleosomal AP surfaces and enables cGAS mobilization, cytoplasmic relocalization and activation by dsDNA, either from pathogens or host itself.

### 4.3 Unclustered

Our biological functional analysis showed that IFIX, LRRFIP1, hnRNPA2B1 and DDX41 exist as an individual cluster, suggesting their distinct functions. To simplify the description, we grouped them together and called it unclustered (**Figure 1D**).

#### 4.3.1 IFIX

IFIX, another PYHIN family member, contains an N-terminal PYD domain and a single DNA-binding HIN domain. Though similar to IFI16 structurally, IFIX has different biological functions and is often associated with promyelocytic leukemia (PML) nuclear bodies. It is primarily localized in the nucleus but can translocate to the cytoplasm under specific conditions, such as during viral infection or in response to specific cellular signals (195, 196). This translocation is regulated by the acetylation of its NLS motifs, with acetylation at K138 in the NLS2 motif promoting shuffling from the nucleus to the cytosol during viral infection (196). Like other PYHIN/ALR family members, IFIX recognizes DNA viruses such as HSV-1 and VACV, binding dsDNA through its HIN domain (195), eliciting an antiviral response (195, 196). Proteomic studies using AP-MS revealed that IFIX interacts with components of PML bodies and DNA damage response effectors, displaying its role in antiviral defense (195). Additionally, nuclear and cytoplasmic proteomic analysis during HSV-1 and VACV infections suggest that IFIX upregulates proteins involved in immune signaling and responses (196).

#### 4.3.2 LRRFIP1

Leucine-Rich Repeat Flightless-Interacting Protein 1 (LRRFIP1), also known as GC-binding factor 2 (GCF2), is a multifunctional protein involved in transcriptional repression, cytoskeletal organization, signal transduction, and immune responses. It consists of three domains: an N-terminal helix domain, a central coiled-coil domain and a C-terminal nucleic acid binding domain (**Figure 1A**). LRRFIP1 functions as a nucleic acid sensor, recognizing dsRNA from vesicular stomatitis virus and dsDNA from *Listeria monocytogenes* (197). All three domains are believed to contribute to its nucleic acid-binding ability (198). A recent study has shown that reduced LRRFIP1 protein level correlates with the severity of SARS-CoV-2 infection and LRRFIP1 suppresses SARS-CoV-2 infection in a type I IFN-independent manner (199). Another study found that patients with severe COVID-19 exhibit increased alternative splicing of the *LRRFIP1* transcript, resulting in reduced levels of functional protein and increased levels of truncated isoforms, suggesting a correlation between perturbed *LRRFIP1* splicing and the severity of SARS-COV-2 infection (200). However, the precise mechanisms by which LRRFIP1 suppresses SARS-CoV-2 infection remain unknown.

#### 4.3.3 hnRNPA2B1

Human hnRNPA2B1 is a small yet significant protein. It consists of 353 amino acids and belongs to the A/B subfamily of ubiquitously expressed heterogeneous nuclear ribonucleoproteins (hnRNPs), a family well known for its roles in RNA processing. Structurally, hnRNPA2B1 contains two RNA recognition motifs (RRM1 and RRM2) in its N-terminus, along with an RGG box and a NLS at its C-terminus (**Figure 1A**). Dr. Xuetao Cao’s group used biotinylated genomic DNA of HSV-1, coupled with 2D SDS–PAGE and mass spectrometry, and identified hnRNPA2B1 as a sensor for HSV-1 DNA (201). Further analysis revealed that two RRM domains are responsible for binding HSV-1 DNA. This discovery suggests a broader role for hnRNPA2B1 in nucleic acid sensing and immune signaling.

#### 4.3.4 DDX41

DDX41 is a member of the DEAD-box family helicases within Superfamily 2 (SF2) and contains the conserved DEAD (Asp-Glu-Ala-Asp) sequence in motif II. Different from DEIH-box helicase DHX9 and DHX36, DDX41 exists as a separate cluster. Predominantly located in the nucleus, DDX41 plays versatile roles in RNA metabolism and the innate immune response. Notably, DDX41 serves as a PRR, detecting the dsDNA from HSV-1 (202, 203) and RNA: DNA hybrids derived from the retrovirus murine leukemia virus (MLV) (204).

The molecular mechanisms underlying DDX41’s recognition of viral DNA and its initiation of antiviral responses have been extensively studied. Research by Liu’s group demonstrated that motifs I (Walker A) and II (Walker B, containing the DEAD sequence) in DDX41 are essential for viral DNA recognition and for its interaction with STING, initiating type I IFN production (202). Following the binding of pathogenic dsDNA or RNA: DNA hybrids, DDX41 translocates from the nucleus to the cytosol, where it colocalizes with STING and activates the STING-TBK1-IRF3 and NF-κB pathways, leading to the induction of type I IFNs and proinflammatory cytokines (202–205). Recent findings from our group have confirmed that DDX41 acts as a viral sensor upstream of cGAS, triggering cGAS-STING-TBK1 to induce type I IFN production in response to dsDNA viruses, such as HSV-1 (203). Though these findings highlight the importance of DDX41 in antiviral signaling, the exact location(s) of viral DNA recognition by DDX41 remains unclear.

## 5 Viral strategies to evade human immune sensors

The interaction between humans and viruses represents a dynamic, ongoing evolutionary arms race. Both the human host and the virus continuously adapt to each other’s strategies, driving co-evolution. As humans develop more effective immune defenses, viruses evolve increasingly sophisticated evasion mechanisms. Below are some general strategies employed by intracellular viruses to evade detection and interfere with host cell signalling cascades.

### 5.1 Sequestration of viral nucleic acids

From entry to exit, viral NAs are released into the cytoplasm or nucleus, where they undergo transcription, translation, and replication; steps that expose them to host NA sensors. To evade this immune surveillance, many viruses sequester their NAs within viral replication compartments or vesicles. For example, the nucleocapsid (N) protein of SARS-CoV-2 binds to the viral RNA, forming ribonucleoprotein (RNP) complexes (206, 207). This binding helps shield the viral RNA from being detected by host sensors like RIG-I and MDA5. Moreover, coronaviruses such as SARS-CoV-2 replicate and transcribe their RNA within double-membrane vesicles (DMVs), a process facilitated by a pore complex formed by NSP3 and NSP4 (208, 209). This complex enables the newly synthesized viral RNA to exit the DMV into the cytoplasm while preserving vesicle integrity, thereby minimizing the exposure of viral RNA to immune sensors. HIV-1 carries out reverse transcription and replication of its genome inside its capsid, which contains selective pores to allow entry of nucleotides from the host cell, while shields the viral DNA from recognition by cGAS (210). WNV and ZIKV also form replication complexes within membrane-bound compartments. These structures help sequester viral RNA, preventing recognition by PRRs. Picornaviruses, including PV, hijack intracellular membranes to create replication organelles. These organelles provide a protected environment for viral RNA synthesis. Kaposi’s sarcoma-associated herpesvirus (KSHV) ORF52 protein oligomerizes and binds to dsDNA, thereby inhibiting the association between DNA and cGAS (211). HSV can enter a latent state within neural ganglia, where its genome is maintained as an episome in the host cell nucleus (212). During latency, viral gene expression is minimized, aiding immune evasion by cGAS and IFI16 present in neurons.

### 5.2 Modification of viral nucleic acids

Viruses can modify their NAs to evade detection by host sensors. These include capping, methylation, RNA editing, RNA cleaving, and RNA decay:

#### 5.2.1 Capping

The 5’ cap is a modified guanosine, with a methyl group added to the 7-position (7-methylguanosine, m7G) at the 5’ end of RNA. The 5’ cap is a key feature found in eukaryotic mRNAs that enhances their stability and translation efficiency. Many viruses adopt this 5’ cap for their RNAs to mimic host mRNA, thereby evading detection by host PRRs like RIG-I. Notable examples include SARS-CoV-2 (213), influenza virus (214), and flaviviruses like DENV (215) and WNV (216).

#### 5.2.2 Methylation

Some viruses methylate their RNA to prevent recognition by host sensors. For example, SARS-CoV-2 uses its nsp16 protein to methylate the 2′-O position of the ribose in the first nucleotide of its RNA, thereby helping the virus evade the host immune response (217). Similarly, the N6-methyladenosine (m^6^A) modification in HBV and hepatitis C virus (HCV) transcripts prevents recognition by RIG-I (218).

#### 5.2.3 RNA editing

Viruses can change their RNA sequences through processes like adenosine-to-inosine (A-to-I) editing. This editing can alter the viral RNA in a way that reduces its recognition by host sensors. Many viruses, including Hepatitis Delta virus (HDV) and Measles virus (219), employ this strategy.

#### 5.2.4 RNA cleaving

The NSP15 protein of SARS-CoV-2 cleaves viral RNA at specific sites, particularly at uridine residues, thereby preventing dsRNAs accumulation, which otherwise be recognized by sensors like MDA5, OAS, and PKR (220).

#### 5.2.5 RNA decay

ZIKV and DENV form RNase L-induced bodies that promote the degradation of viral RNA (221).

Notably, DNA viruses do not typically use DNA editing mechanisms in the same way that RNA viruses do.

### 5.3 Integration of viral nucleic acids into human genome

Viruses can integrate their NAs into the host genome to evade host cell sensors and establish persistent infections. The best example of this strategy is retroviruses, such as HIV. Upon entering CD4+ T cells, HIV uses its reverse transcriptase to convert its ssRNA into dsDNA within the capsid as it travels through the cytoplasm towards the nucleus. The newly synthesized viral DNA, along with associated viral proteins (including integrase), forms a complex known as the pre-integration complex, which is transported into the host cell nucleus. The viral enzyme integrase catalyzes the integration of the viral DNA into the host cell genome through a “cut-and-paste” process, resulting in the formation of the provirus (222). This integration allows HIV to establish latency and evade immune detection. Other retroviruses, such as HTLV, utilize the same strategy.

DNA viruses, including human papillomavirus (HPV), HBV, and human herpesvirus 6 (HHV-6), can also integrate their DNA into the host genome. HPV does so particularly in the case of high-risk strains associated with cervical cancer, where integration disrupts normal cellular functions and can lead to oncogenesis (223). HBV can integrate segments of its DNA into the host genome, promoting chronic infection and increasing the risk of liver cancer (224). HHV-6 can integrate its DNA into the telomeres of human chromosomes (225), enabling the virus to persist in a latent state and evade immune detection.

These integration events promote long-term persistence within the host—often lasting a lifetime—and allow the virus to remain latent and reactivate under certain conditions.

### 5.4 Inhibition and degradation of human sensors

Viruses have evolved various strategies to inhibit or degrade host sensors and evade immune surveillance. These strategies target multiple levels of host defense, including DNA, RNA, transcription, translation, protein stability and function, as well as post-translational modifications (PTMs).

Some viruses produce specific proteins that directly suppress host sensors. For example, influenza A virus produces the NS1 protein, whose E96/E97 residues mediate interaction with the coiled-coil domain of TRIM25, inhibiting TRIM25 multimerization and the ubiquitination of RIG-I CARD domain, ultimately suppressing RIG-I signal transduction (226). The VP35 protein of Ebola virus inhibits RIG-I activation by sequestering viral RNA to prevent the induction of an IFN response (227). The V protein of paramyxoviruses interacts with the helicase domain of MDA5, disrupting its proper folding and preventing the formation of filament structures required for activation (228). The US11 protein of HSV-1 binds and inhibits PKR activation (229), while also binding dsRNA, sequestering it from OAS, and inhibiting OAS activation (230).

Moreover, some viruses can degrade host sensors at the mRNA or protein level. At the RNA level, EBV encodes the miRNA BART6-3p, which targets the 3′UTR of RIG-I mRNA, leading to its degradation, suppressing downstream RIG-I signaling and reducing the induction of type I IFN and ISGs (231). SARS-CoV nsp1 promotes the degradation of host mRNAs (232). Ribonuclease L (RNase L) degrades host mRNAs during DENV or SARS-CoV-2 infection (233). Notably, RNase L degrades both viral RNA and host mRNA, with its activity being context-dependent and tightly regulated. At the protein level, HSV-1 encodes the E3 ubiquitin ligase ICP0, which targets and degrades IFI16 (234). EMCV uses its VP2 protein to degrade IFI16 through a caspase-dependent apoptosis pathway (235). Moreover, some viruses can inhibit the translation of host genes. For instance, HIV-1 produces its accessory protein Vpr, which affects the phosphorylation/activity of the translation initiation factor 4E (eIF4E), thus inhibiting the translation of host mRNAs (236).

## 6 Antiviral drug and vaccine development targeting nucleic acid sensing

Approximately 40 million HIV-1 patients are currently receiving antiretroviral therapies (ART) to sustain their health and prolong survival (237). COVID-19 vaccines have been credited with saving 14.4 million lives across 185 countries and territories during the pandemic in 2020 and 2021 (238). However, new viral strains continue to emerge due to genetic mutations, recombination, and environmental changes, as demonstrated by recent human cases of H5N1 avian influenza. As of June 2025, both Canada and the United States are experiencing significant measles outbreaks. Therefore, it is crucial to be prepared for future viral threats. Various strategies have been employed in drug discovery and development to target virus or host cellular factors, focusing on viral nucleic acids and host sensors especially.

### 6.1 Targeting viral nucleic acids

Targeting viral nucleic acids has emerged as a powerful strategy for antiviral drug development, particularly with advances in nucleic acid–based technologies. Various approaches are employed to combat viral diseases, including nucleoside analogs, antisense oligonucleotides (ASOs), aptamers, siRNA, and CRISPR-based systems.

Nucleoside analogs have long been utilized as antiviral drugs. By competing with natural nucleotides, they are incorporated into the growing viral DNA or RNA strand, disrupting viral replication. For SARS-CoV-2, remdesivir (239, 240), an adenosine analog, and molnupiravir (241, 242), a cytidine analog, have demonstrated effective inhibition of viral replication. Both drugs received U.S. FDA approval in 2020 and 2021, respectively, as standard treatments for COVID-19. For HIV, zidovudine (243), a thymidine analog, was the first drug approved in 1987. It is metabolized to its active form, zidovudine triphosphate (ZDV-TP), which, upon incorporation into viral DNA by reverse transcriptase, terminates DNA chain elongation and halts viral replication. Due to toxicity concerns, zidovudine is now rarely used. Several newer nucleoside analogs have since been developed and are currently in use, including lamivudine and emtricitabine (both cytidine analogs), abacavir (a deoxyguanosine triphosphate mimic), and tenofovir disoproxil fumarate and tenofovir alafenamide (both adenosine monophosphate mimics) (244). Nucleoside analogs are also widely applied to other viral infections, such as acyclovir (a guanosine analog) for HSV, HBV, and ribavirin (a guanosine analog) and sofosbuvir (a uridine analog) for HCV.

Antisense oligonucleotide (ASO) techniques utilize single-stranded DNA or RNA molecules that bind specifically to viral mRNA, resulting in its degradation or inhibition of translation. Fomivirsen, developed by Isis Pharmaceuticals and approved by the FDA in 1998, is a 21-base phosphorothioate oligonucleotide that targets the immediate-early gene mRNA of human cytomegalovirus (HCMV), thereby inhibiting viral replication by preventing the synthesis of essential viral proteins (245). Aptamers are structured nucleic acids that bind to viral proteins or RNA, blocking viral entry or replication. Although no aptamer-based drugs for viral diseases have yet received FDA approval, several candidates have demonstrated strong potential in preclinical and early clinical studies, such as anti-gp120 aptamers targeting HIV (246). siRNA approaches, based on RNA interference (RNAi) mechanisms, involve synthetic double-stranded RNA molecules designed to target and degrade viral RNA (247). These strategies have been explored for viruses including HBV, SARS-CoV-2, HIV, and influenza viruses. CRISPR-based antiviral methods employ RNA-guided nucleases, such as Cas9 for double-stranded DNA and Cas13 for single-stranded RNA, to selectively degrade viral genetic material. This approach has been investigated for viruses such as HSV, HIV, SARS-CoV-2, and HBV (248). While siRNA and CRISPR-based antiviral therapies hold considerable promise, they face significant challenges including off-target effects, delivery efficiency, and potential immune responses.

### 6.2 Targeting host nucleic acid sensors

Due to the continuous mutation of viruses, host-targeting drugs offer advantages over virus-targeting therapies by maintaining efficacy through action on conserved cellular factors. Multi-omics studies combined with computational analyses have identified host dependency factors, including host nucleic acid sensors, and assessed their druggability and therapeutic potential. Therapeutic strategies focus either on enhancing antiviral immunity (e.g., in chronic infections) or suppressing excessive inflammation (e.g., in viral sepsis or cytokine storms).

To stimulate antiviral immunity, agonists targeting TLR7 and RIG-I have been developed. Vesatolimod (GS-9620), a synthetic small molecule developed by Gilead Sciences, functions as a TLR7 agonist that enhances innate immunity by inducing type I interferons and other cytokines. Vesatolimod is currently in Phase 2a clinical trials, with previous studies demonstrating its ability to reduce latent viral reservoirs in HIV (249) and hepatitis B infections (250). To mimic viral RNA, synthetic double-stranded RNA molecules with 5′-triphosphate ends have been designed to activate RIG-I. RGT100 (MK-4621) (251), developed by Rigontec (acquired by Merck in 2017), is in Phase I/II trials for advanced solid tumors and lymphomas (NCT03065023, NCT03739138). RIG-101, developed by RIGImmune, is in preclinical development for respiratory viruses such as influenza, RSV, and rhinovirus (252). TTX-RIGA, by TransCode Therapeutics, is in preclinical trials aimed at stimulating immune responses within the tumor microenvironment.

Conversely, excessive immune activation can cause severe tissue damage, chronic inflammation, and mortality. Accordingly, antagonists or inhibitors targeting host sensors have been developed to suppress hyperinflammation. VENT-03, a cGAS antagonist developed by Ventus Therapeutics, has shown efficacy in reducing inflammation and autoimmune responses in diseases such as lupus, dermatomyositis, and systemic sclerosis (253) and is currently in Phase 2 trials. Pharmacological inhibitors targeting the NLRP1 inflammasome, including Curcumin (254), Propofol (255), and HY−021068 (256), have demonstrated beneficial effects in animal models. Inhibitors of DDX41 have also been identified (257), though no clinical trials have been reported to date. It is important to note that host nucleic acid sensors have diverse functions beyond viral detection and are implicated in various diseases and cancers. Continued molecular and cellular mechanistic studies integrating biochemical, multi-omic, and computational approaches will advance our understanding of host factor-mediated processes and facilitate the development of improved therapeutic options for viral and other diseases.

### 6.3 Vaccine development

Based on the RNA sequence of SARS-CoV-2, mRNA vaccines were rapidly developed by Pfizer-BioNTech and Moderna during the pandemic (258). These vaccines contain synthetic mRNA encoding the SARS-CoV-2 spike protein, encapsulated within lipid nanoparticles to protect the mRNA and facilitate its delivery into human cells. Upon vaccination, host cells express the spike protein, which elicits adaptive immune responses from both B and T cells, resulting in the formation of memory cells that confer long-lasting immunity and protection against future infections. These vaccines not only represent the first widespread use of mRNA technology in humans but also demonstrate its effectiveness, safety, and scalability on a global scale. mRNA vaccines are currently under development for other viral diseases, including HBV, RSV, ZIKV, Ebola, HPV, and DENV.

Although DNA vaccines are more stable and easier to manufacture than mRNA vaccines, they carry a higher risk of integration into the host genome, which may lead to tumorigenesis. ZyCoV-D, approved in India in 2021, is the first DNA vaccine authorized for human use against COVID-19 (259). It consists of a circular plasmid DNA encoding the SARS-CoV-2 spike protein along with an IgE signal peptide. While ZyCoV-D remains the only DNA vaccine approved for human use to date, several others are in development or undergoing clinical trials targeting HIV, ZIKV, Ebola, influenza, and HPV. Challenges such as low immunogenicity in humans and the difficulty of delivering plasmid DNA into the host cell nucleus continue to hinder DNA vaccine development, highlighting the need for further research.

Viral vector-based vaccines have been developed due to their higher delivery efficiency and immunogenicity. ChAdOx1 nCoV-19, also known as AZD1222, is a COVID-19 vaccine developed by AstraZeneca (260). It employs a replication-deficient chimpanzee adenovirus vector (ChAdOx1) to express the SARS-CoV-2 spike (S) protein. For Ebola virus, the rVSV-ZEBOV vaccine (Ervebo), developed by Merck, utilizes VSV to express the glycoprotein of Zaire ebolavirus, replacing its own envelope protein. Viral vector-based vaccines are also under development for HIV and ZIKV.

## 7 Concluding remarks and future perspective

As depicted at the conclusion of the film *Catch Me If You Can*, Frank Abagnale was arrested in France, extradited to the United States, and sentenced to 12 years in prison. Notably, due to his exceptional talents and skills, he later collaborated with the FBI and became a leading expert in bank fraud. His work with the FBI significantly contributed to strengthening security systems, ultimately fostering a safer financial environment for society. Similarly, host cells employ a variety of strategies to detect viral invasion and suppress viral replication. In response, viruses continuously evolve mechanisms to evade detection, inhibit cellular antiviral responses, and hijack host cellular machinery for their replication. This ongoing co-evolutionary arms race results in constant adaptation between viruses and their hosts. So far we know host nucleic acid sensors for virus:

- To date, research has identified a total of 26 human sensors for viral nucleic acids, exhibiting remarkable diversity. These sensors possess distinct domains for viral nucleic acid binding and are distributed across various cellular compartments, including the cytoplasm, nucleus, and endosomes. Some sensors specifically bind DNA, others RNA, while several recognize both nucleic acid types.
- Viruses employ multiple strategies to evade these immune sensors.
- Dysregulation of viral nucleic acid sensing pathways can lead to autoimmunity, autoinflammation, and various diseases, including cancers.
- Drugs and vaccines have been developed targeting human viral nucleic acid sensing pathways. Following the successful deployment of COVID-19 mRNA vaccines, vaccines based on mRNA and DNA platforms for RSV, influenza, HIV, HPV, HCV, and other viral diseases are either already in use or under development. Both agonists and antagonists targeting host immune sensors are actively being explored as therapeutic strategies.

To enhance our ability to prevent and combat viral infections and thereby sustain human and animal health, it is essential to deepen our understanding of viral molecules and host factors. In advancing antiviral drug discovery and development, researchers have increasingly emphasized the application of multi-omics approaches to elucidate the molecular mechanisms governing the complex interactions between viruses and their hosts. Key future areas of focus in antiviral treatment development include:

- High-throughput screening technologies: Advanced high-throughput methods, such as CRISPR-based screens, generate large datasets that improve our understanding of virus-host interactions. These technologies facilitate the identification of potential therapeutic targets and provide insights into the molecular mechanisms underlying viral infections.
- Artificial intelligence (AI): AI is poised to revolutionize antiviral drug discovery by accelerating and optimizing multiple stages, including data analysis and mining, drug design and virtual screening, natural language processing, drug repurposing, predictive modeling, and clinical trial optimization.
- Host-pathogen interaction networks: Researchers are developing systematic frameworks to map the complex networks of interactions between hosts and pathogens. This includes identifying critical host factors exploited by viruses and understanding how these interactions affect disease progression and outcomes.
- Understanding viral evolution: Investigating viral evolution and adaptation to host defenses is crucial for predicting and preventing future pandemics. Such insights promote public health strategies and enhance global preparedness against emerging viral threats.
- Improved diagnostic tools: A more comprehensive knowledge of viral replication and host factors enables the development of more accurate and rapid diagnostic tests. Early detection facilitates timely interventions and helps limit viral transmission.
- Personalized medicine: Enhanced understanding of host-virus interactions allows for treatments tailored to individual patients based on genetic profiles and immune responses, including PRRs and PAMPs. This personalized approach can improve therapeutic efficacy and reduce adverse drug effects.

## Glossary

ACE2: angiotensin-converting enzyme 2
AdV: adenovirus
AIM2: Absent in melanoma 2
APCs: antigen-presenting cells
BP: Biological process
BPEV: Bell Pepper Endornavirus
CARD: Caspase activation and recruitment domain
CCR5: C-C chemokine receptor type 5
cGAS: cyclic GMP-AMP synthase
CTD: C-terminal domain
CV: Coxsackie virus
DCs: Dendritic cells
DDX41: DEAD-box helicase 41
DENV: Dengue virus
DMV: double-membrane vesicle
DNA-PK: DNA-dependent protein kinase
dsDNA: double-stranded DNA
dsRBD: double-stranded RNA binding domain
EBV: Epstein-Barr virus
EBOV: Ebola virus
EMCV: Encephalomyocarditis virus
ENV: envelope
EV: enterovirus
GO: Gene ontology
HBV: hepatitis B virus
HCMV: human cytomegalovirus
HCV: hepatitis C virus
HHV: human herpesvirus
HIV: human immunodeficiency virus
HPV: human papilloma virus
HSCs: hematopoietic stem cells
HSV-1/2: herpes simplex virus 1/2
HTLV: human T-cell lymphotropic virus
IAV: influenza A virus
IFI16: interferon gamma inducible protein
IFNs: interferons
ISGs: interferon-stimulated genes
JEV: Japanese Encephalitis Virus
KSHV: Kaposi’s sarcoma-associated herpesvirus
LGP2: Laboratory of genetics and physiology 2
LRR: Leucine-rich repeat
LRRFIP1: Leucine-rich repeat flightless-interacting protein 1
MAVS: mitochondrial anti-viral-signaling protein
MARV: Marburg virus
MCMV: murine cytomegalovirus
MDA5: melanoma differentiation-associated protein 5
MHV: mouse hepatitis virus
MLV: murine leukemia virus
MVA: modified vaccinia ankara
MV: measles virus
NA: nucleic acid
NDV: newcastle disease virus
NES: nuclear export signal
NLRP1: NBD-, LRR- and Pyrin domain-containing protein 1
NLRP6: NBD-, LRR- and Pyrin domain-containing protein 6
NLS: nuclear localization signal
NHEJ: non-homologous end joining
NK: natural killer
OAS: 2′-5′-Oligoadenylate synthetases
OB: oligonucleotide/oligosaccharide-binding
PV: poliovirus
PAMPs: pathogen-associated molecular patterns
PAZ: Piwi-Argonaute-Zwille
PKR: protein kinase R
PML: promyelocytic leukemia
PPRs: pattern recognition receptors
RdRp: RNA dependent RNA polymerase
RBD: RNA binding domain
RIG-I: retinoic acid-inducible gene-I
RLRs: retinoic acid-inducible gene (RIG)-I-like receptors
RNAi: RNA interference
RRM: RNA recognition motif
RSV: respiratory syncytial virus
SFV: semliki forest virus
SGs: stress granules
SINV: Sindbis virus
siRNA: small interfering RNA
ssDNA: single-stranded DNA
STING: Stimulator of interferon genes
SeV: Sendai virus
TIR: Toll/Interleukin-1 Receptor domain
TLR: Toll like receptor
UPA: UNC5, PIDD and Ankyrin domain
VACV: vaccinia virus
VSV: vesicular stomatitis virus
VZV: Varicella-Zoster virus
WNV: West Nile virus
ZAP: Zinc-finger antiviral protein
